# MTA2-mediated inhibition of PTEN leads to pancreatic ductal adenocarcinoma carcinogenicity

**DOI:** 10.1038/s41419-019-1424-5

**Published:** 2019-02-27

**Authors:** Wenzhe Si, Xujun Liu, Rui Wei, Yuan Zhang, Yang Zhao, Liyan Cui, Tianpei Hong

**Affiliations:** 1 Department of Laboratory Medicine, Peking University Third Hospital, 100191 Beijing, China; 2 Key Laboratory of Carcinogenesis and Translational Research (Ministry of Education), Department of Biochemistry and Molecular Biology, School of Basic Medical Sciences, Peking University Health Science Center, 100191 Beijing, China; 3 Department of Endocrinology and Metabolism, Peking University Third Hospital, 100191 Beijing, China

## Abstract

Metastasis-associated protein 2 (MTA2) is a core subunit of the nucleosome remodeling and deacetylating (NuRD) complex and functions by mediating chromatin remodeling and gene silencing. However, its biological actions and clinical significance in pancreatic ductal adenocarcinoma (PDAC) remain elusive. The aim of this study was to explore the function and regulation mechanism of MTA2 in PDAC. As shown in GEO, ICGC, and TCGA databases, a higher expression of MTA2 was noticed in the PDAC tissues than in the normal pancreatic tissues. Moreover, a higher expression level of MTA2 was associated with a shorter overall survival time in these public PDAC databases. We further investigated the underlying mechanisms of these observations by using a chromatin immunoprecipitation (ChIP)-based deep sequencing, luciferase reporter, and quantitative ChIP assays. We identified the repressive binding of MTA2 to the promoter of *phosphatase and tensin homolog* (*PTEN*). We also found that Snail recruited MTA2 and HDAC1 to suppress PTEN expression. Ectopic expression and knockdown of *MTA2* were performed to evaluate the effects of this gene on PDAC cell proliferation, migration, and invasion. Using CCK-8, colony formation and transwell assays, and a xenograft tumor model, we revealed that MTA2 promoted PDAC cell proliferation, migration, and invasion in vitro and PDAC tumor growth in vivo by downregulation of PTEN. In benzyl isothiocyanate (BITC)-treated MIA Paca-2 cells and PANC-1 cells, MTA2 level decreased in a dose- and time-dependent manner with concomitant upregulation of PTEN level and downregulation of phosphorylated PI3K and AKT levels, providing evidence of the involvement of MTA2 and PTEN in the regulation of the PI3K/AKT pathway in BITC-mediated PDAC suppression. Collectively, these findings uncover a novel role for MTA2 in the regulation of PDAC progression and help to elucidate the mechanisms involved in this process.

## Introduction

Pancreatic cancer, which causes an estimated 227,000 deaths per year, is one of the most lethal malignancies worldwide and has a 5-year survival rate of <5%^1–3^. The most common histological type of pancreatic cancer is pancreatic ductal adenocarcinoma (PDAC), which presents at an advanced stage, has a highly metastatic and markedly chemo-resistant phenotype and is responsible for an extremely poor clinical prognosis^4–7^. To date, potent and low-toxic medications for the treatment of PDAC patients remains deficient. Hence, in order to improve pharmacotherapy for this disease, it is important to elucidate the molecular mechanisms underlying PDAC cell proliferation and metastasis.

*Phosphatase and tensin homolog* (*PTEN*) is a well-known tumor suppressor gene with diverse functions in many cellular processes, including cell viability, senescence, proliferation, and invasion^8,9^. Heterozygous deletion of *Pten* leads to multiple tumors in mice, whereas homozygous mice results in early embryonic lethality^10,11^, indicating that PTEN plays a pivotal role in various cancer types, including pancreatic cancer^12–14^. Furthermore, the expression of PTEN is downregulated by several mechanisms, including genomic loss, epigenetic silencing and transcriptional repression or negative post-transcriptional regulation, such as phosphorylation, ubiquitination, and acetylation^15–18^. Although PTEN has been extensively studied by different groups in the cancer research field, the regulatory mechanism of PTEN in pancreatic cancer warrants further study.

Metastasis-associated gene 2 (MTA2) is a member of the MTA family and is identified as one component of the nucleosome remodeling and deacetylation (NuRD) complex^19–21^. MTA2 has been shown to modulate gene expression by affecting chromatin remodeling and transcription procedures^22,23^. A higher MTA2 expression is clearly related to a poorer prognosis in cancer patients and is involved in the development and progression of cancer during carcinogenicity^24–26^. To the best of our knowledge, there is one study reporting the high expression pattern of MTA2 in PDAC;^27^ however, the precise function and regulation mechanism of MTA2 has not been documented to date.

Benzyl isothiocyanate (BITC), a compound which is found in cruciferous vegetables and functions as chemoprotective agents against carcinogenesis, is well known to have anticancer properties and to be non-toxic to normal pancreatic epithelial cells. As the pathogenesis of PDAC is complex and characterized by deregulation of multiple checkpoints and activation of several oncogenic pathways, the beneficial effect of BITC in cancer chemoprevention is desirable to target multiple pathways and lacks of target-specificity^28^. However, the mechanism by which BITC inhibits human pancreatic carcinogenesis is not fully understood.

## Results

### A higher expression level of MTA2 predicts a poorer prognosis in patients with pancreatic cancer

**Fig. 1:**
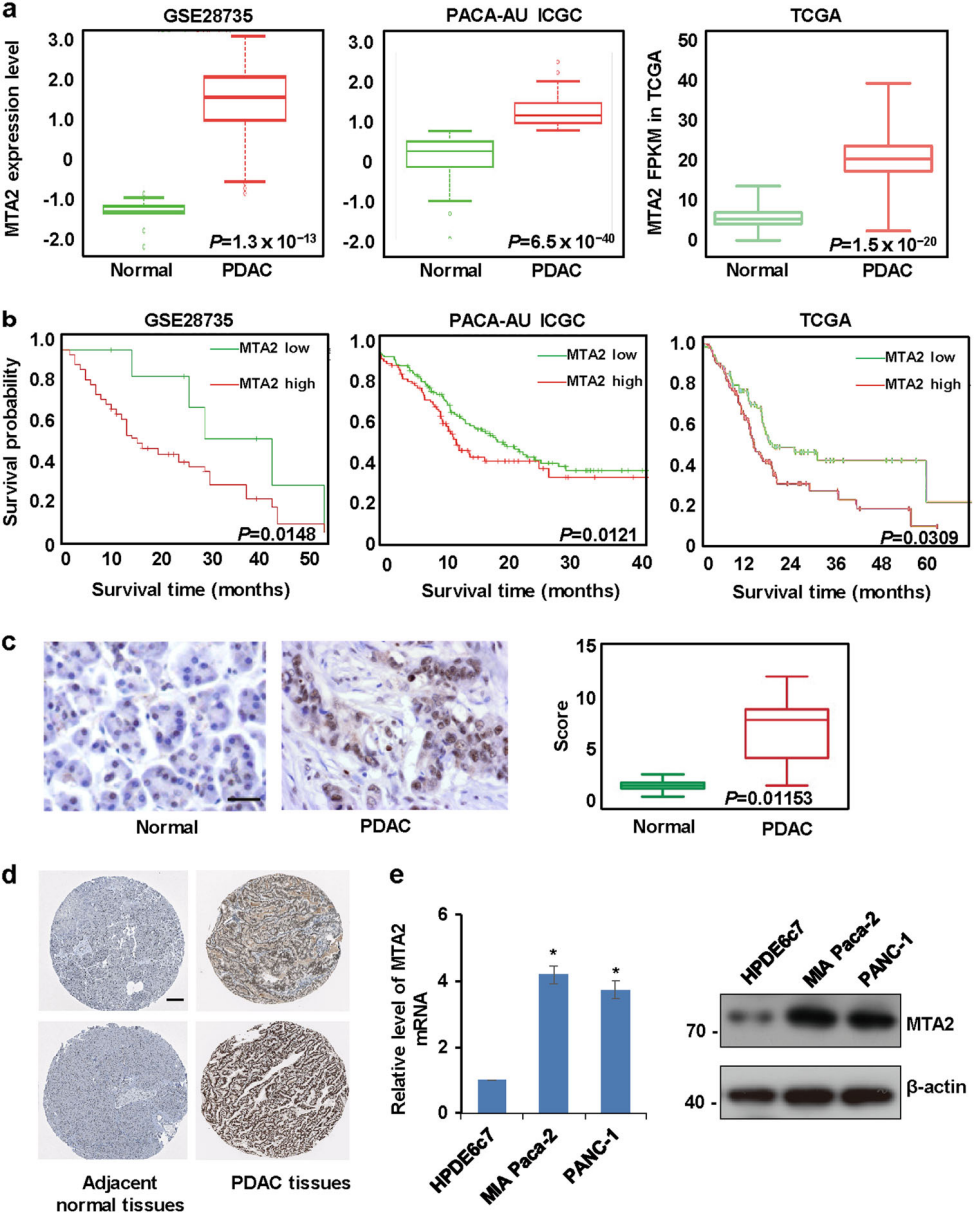
A higher expression level of MTA2 predicts a poorer prognosis of pancreatic cancer. **a** The relative expression of MTA2 was measured in different pancreatic ductal adenocarcinoma (PDAC) cohorts in GSE28735, PACA-AU ICGC, and TCGA databases. **b** The Kaplan–Meier analysis was performed using the GSE28735, PACA-AU ICGC, and TCGA databases, and showed that a higher expression level of MTA2 predicted a poorer overall survival. **c** Tissue microarray (TMA) was used to perform immunohistochemical staining. Representative sections of normal and PDAC tissues stained with anti-MTA2 antibody as well as quantitative immunohistochemistry results of MTA2 expression was presented. **d** The expression of MTA2 was acquired in PDAC specimens and the adjacent normal pancreatic tissues. Images were taken from the online database of the Human Protein Atlas. **e** qRT-PCR and western blot analyses were used to measure the expression of MTA2 in the human pancreatic cancer cell lines MIA Paca-2 and PANC-1 cells, and the human pancreatic duct epithelial cell line HPDE6c7 was included as control. Values are mean ± S.D. *n* = 3. *P < 0.05

It has been demonstrated that MTA2 is associated with aggressive malignant phenotypes of numerous cancers such as breast cancer, hepatocellular carcinoma, and PDAC^29^. Consistently, our analysis using the database of cBioPortal for Cancer Genomics showed that *MTA2* gene was amplified in several types of human cancer, including pancreatic cancer (Supplementary Figure 1). As deferred diagnosis of PDAC is associated with its dismal prognosis, new diagnosis and treatment strategies are urgently required. In this study, we focus our attention on the function of MTA2 in PDAC. By analyzing the Gene Expression Omnibus (GEO), the International Cancer Genome Consortium (ICGC), The Cancer Genome Atlas (TCGA), and the Oncomine databases, we explored the expression of MTA2 in the different PDAC cohorts, and the patients’ information used in this analysis is shown in Supplementary Table 1. We noticed that MTA2 was significantly upregulated in human pancreatic cancer tissues compared with that in non-cancer normal tissues (Fig. 1a and Supplementary Figure 1), and a higher expression level of MTA2 was associated with a shorter overall survival time (Fig. 1b). The TCGA database was used to further analyze the relationship between the clinicopathological parameters and the expression level of MTA2. Higher MTA2 expression level was associated with more advanced AJCC (American Joint Committee on Cancer) stage (Supplementary Table 2).

Next, we detected MTA2 expression in PDAC by tissue microarray (TMA). A series of 64 PDAC tissues and the normal tissues from PDAC patients were collected. Immunohistochemical staining revealed that MTA2 was mainly detected in the nucleus and markedly upregulated in PDAC tissues compared to the adjacent noncancerous tissues (Fig. 1c). Remarkably, high MTA2 expression was correlated with either pathological grade or T stage, while no correlations were found between MTA2 expression level and gender, age, or N stage (Supplementary Table 3). Moreover, we analyzed the MTA2 protein level in clinical specimens from the online database of the Human Protein Atlas. According to Uhlen’s reports^30,31^, MTA2 displayed a positively strong expression in PDAC, and a weak expression in the adjacent normal pancreatic tissues (Fig. 1d). Likewise, our study showed that the expression level of MTA2 was significantly increased in the PDAC cell lines, such as MIA Paca-2, and PDAC-1 cells, compared with the human pancreatic duct epithelial cell line HPDE6c7 (Fig. 1e).

### Identification of potential downstream genes regulated by MTA2 in PDAC cells

**Fig. 2:**
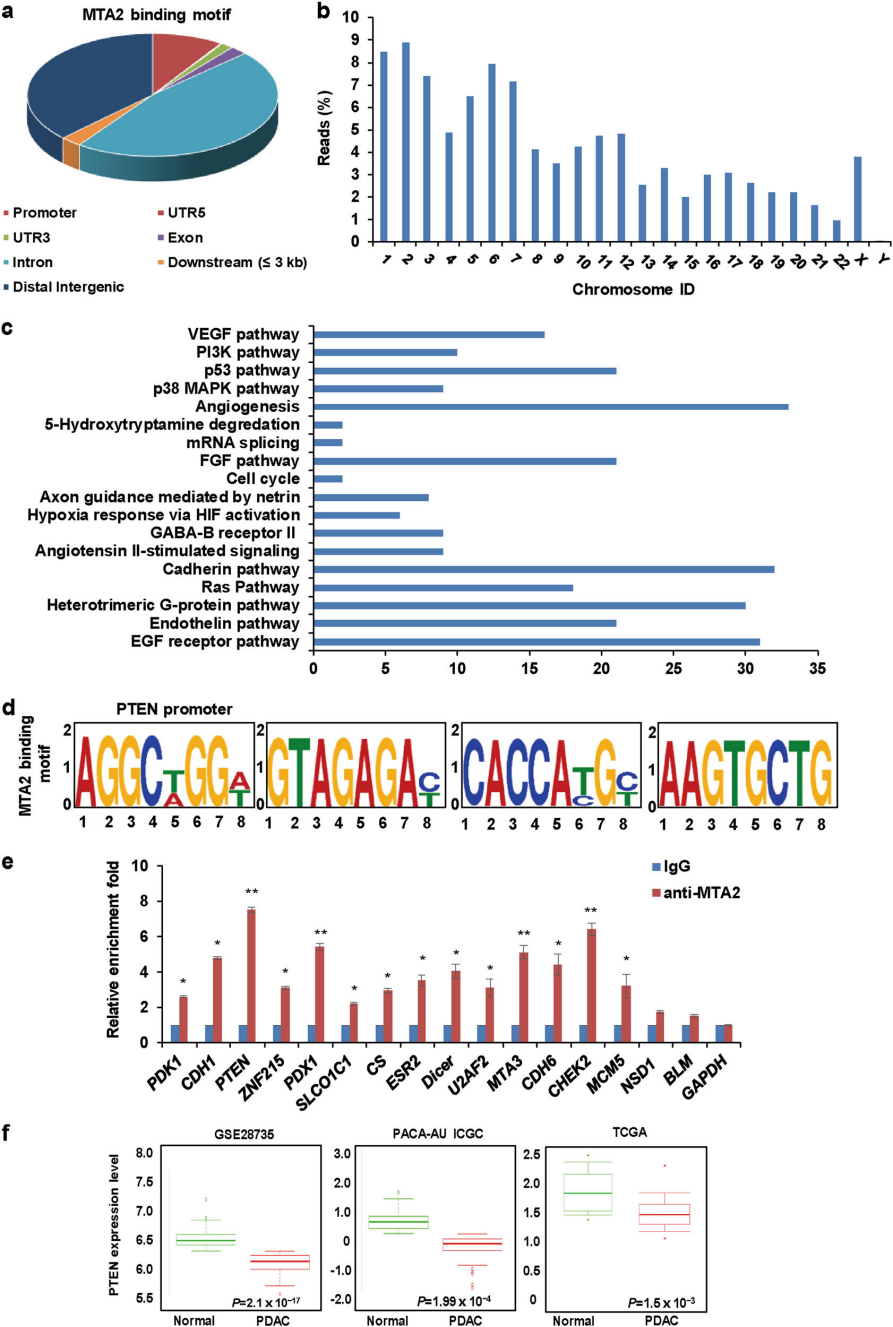
Investigation of downstream genes regulated by MTA2 in PDAC cells. **a** ChIP-seq analysis was performed in MIA Paca-2 cells using a specific antibody against MTA2, and the peaks’ distribution of MTA2 was determined. **b** The relative peaks’ chromosome distribution is shown. **c** KEGG (Kyoto Encyclopedia of Genes and Genomes) pathway database was used to identify the pathways that the MTA2 target genes were involved in. **d** Discriminative Regular Expression Motif Elicitation was used to analyze the binding motif for MTA2. **e** qChIP analysis was performed in MIA Paca-2 cells using anti-MTA2 antibody to detect the binding of MTA2 on the selected target genes. Isotypic IgG served as a control. Data were expressed as fold change over the control. Error bars represent mean ± S.D. for three independent experiments. *P < 0.05; **P < 0.01. **f** The relative expression of PTEN was measured in different PDAC cohorts in GSE28735, PACA-AU ICGC, and TCGA databases

As is known, MTA2 is mainly implicated in the repression of gene transcription. To identify the potential downstream genes regulated by MTA2 in PDAC cells, we analyzed the genome-wide transcriptional targets of MTA2 by a chromatin immunoprecipitation-based deep sequencing (ChIP-Seq). In these experiments, ChIP assays were performed in MIA Paca-2 cells with a specific antibody against MTA2 or an isotypic normal IgG (as a negative control). Following ChIP, MTA2-associated DNAs were amplified using nonbiased conditions, labeled, and sequenced on the HiSeq2000 program. We identified 7371 MTA2-specific binding peaks. The distribution of these peaks was 9.151% promoter, 0.187% UTR5 (5′ untranslated region), 1.615% UTR3 (3′ untranslated region), 2.244% exon, 46.685% intron, 2.755% downstream (≤3 kb), and 37.363% distal intergenic (Fig. 2a). Furthermore, the peaks’ chromosome distribution is shown in Fig. 2b. The genes with the corresponding promoters were then classified into various cellular signaling pathways using the KEGG (Kyoto Encyclopedia of Genes and Genomes) pathway database. With this approach, we identified several pathways that were significantly enriched, such as VEGF (vascular endothelial growth factor), PI3K, P53, and P38 MAPK (mitogen-activated protein kinase) pathways (Fig. 2c). We further used the Discriminative Regular Expression Motif Elicitation to analyze the binding motif for MTA2, which included *PTEN* promoter (Fig. 2d). Quantitative ChIP (qChIP) analysis in MIA Paca-2 cells using a specific antibody against MTA2 or an isotypic normal IgG on the selected genes (including *PTEN*), which represented each of the classified pathways, showed strong enrichments of MTA2 on the promoters of these genes, validating the ChIP-seq results (Fig. 2e). Interestingly, PTEN was significantly downregulated in human pancreatic cancer tissues compared with that in normal tissues in the online public database (Fig. 2f), which displayed an opposite tendency with MTA2.

### *PTEN* is a transcriptional target of the MTA2/NuRD complex in PDAC cells

**Fig. 3:**
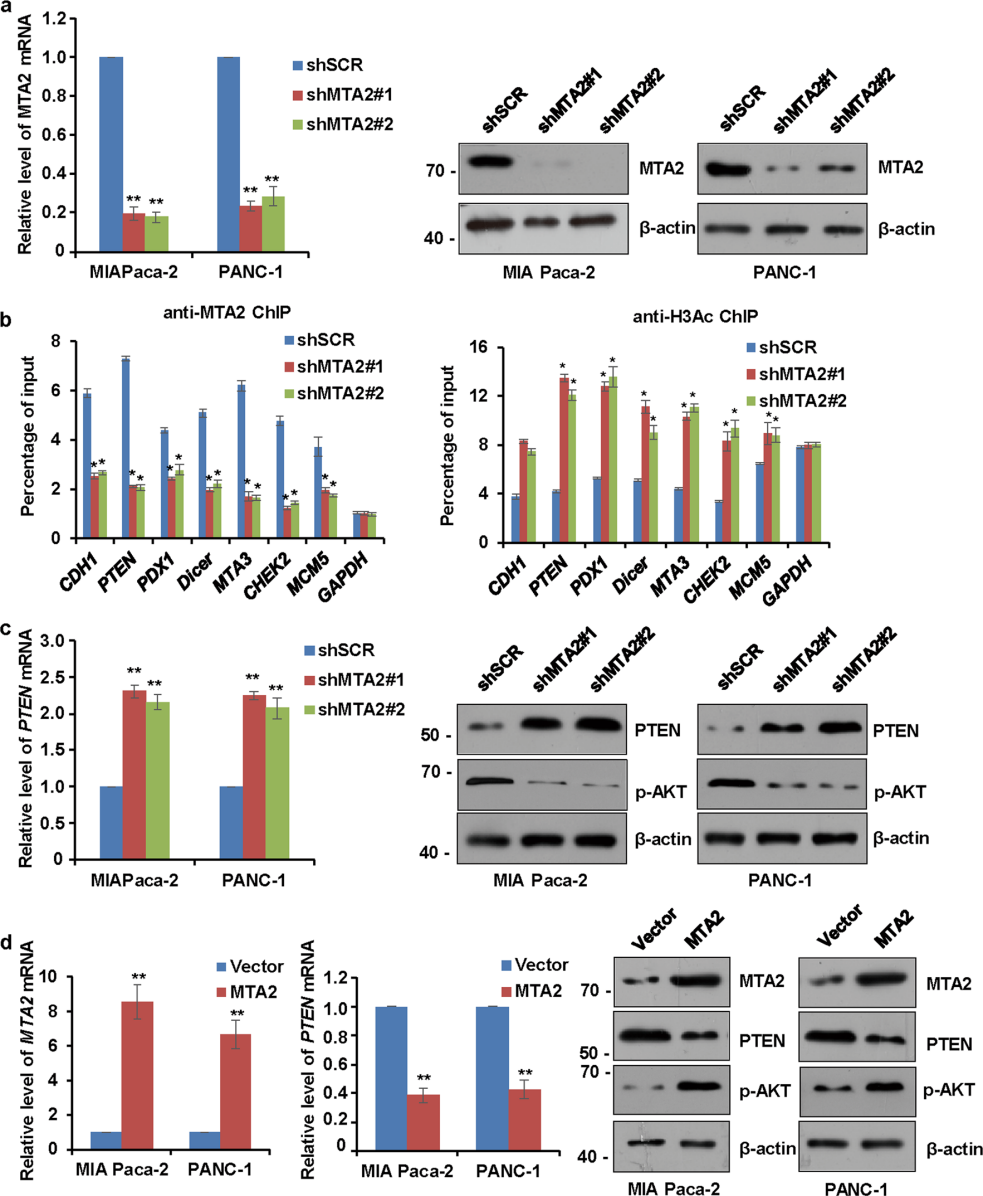
*PTEN* is a transcriptional target of the MTA2/NuRD complex in PDAC cells. **a** MIA Paca-2 or PANC-1 cells were infected with a lentivirus carrying the scrambled control shRNA (shSCR) or shRNAs targeting *MTA2* (shMTA). The knockdown efficiencies of *MTA2* were verified by qRT-PCR and western blot. Values are mean ± S.D. *n* = 3. **P < 0.01. **b** qChIP experiments were performed in the *MTA2*-depleted MIA Paca-2 cells to measure the recruitment of MTA2 (left panel) and H3Ac (right panel) at the promoters of the target genes. Error bars represent mean ± S.D. *n* = 3. *P < 0.05; **P < 0.01. **c, d** qRT-PCR and western blot analyses were used to measure the levels of PTEN and phosphorylated AKT (p-AKT) in MIA Paca-2 or PANC-1 cells infected **c** with shSCR or shMTA2, or **d** with empty vector or *MTA2* overexpression construct. Values are mean ± S.D. *n* = 3. **P < 0.01

To support the notion that MTA2 occupies the target promoters in the context of the MTA2/NuRD complex, MIA Paca-2 cells cloned with stable *MTA2* depletion were generated by lentivirus-delivered specific shRNA (Fig. 3a). qChIP experiments indicated that a depletion of *MTA2* resulted in a marked reduction of the recruitment of MTA2 at the promoters of the target genes (Fig. 3b, left panel). In concert with this finding, the levels of pan-H3 acetylation (H3Ac) on the target genes were significantly increased in the stable *MTA2* knockdown cells (Fig. 3b, right panel), since histone deacetylase 1 (HDAC1) and HDAC2 catalyzed protein deacetylation work as core subunits of NuRD complex with enzymatic functions. The above data indicated that the enrichment of MTA2 was dependent on the NuRD complex. Among these target genes, *PTEN* has been reported to be one of the most powerful tumor repressor gene in numerous cancer types including PDAC^32,33^. Therefore, we explored whether PTEN was functionally linked to the downstream target of MTA2. As shown in Fig. 3c, the depletion of *MTA2* led to an increased expression of PTEN at both mRNA and protein levels in either MIA Paca-2 cells or PANC-1 cells. On the contrary, an overexpression of *MTA2* using recombinant lentiviruses resulted in a decreased expression of PTEN at both transcriptional and translational levels (Fig. 3d). Considering the potential role of PTEN in regulating the PI3K/AKT pathway in the cancer cells, we next determine if the MTA2-mediated PTEN inhibition might affect PI3K/AKT signaling. As expected, knockdown of *MTA2* reduced the level of phosphorylated AKT (p-AKT) protein (Fig. 3c), while overexpression of *MTA2* increased the p-AKT protein level (Fig. 3d).

### Repression of PTEN by MTA2 is dependent on Snail in PDAC cells

**Fig. 4:**
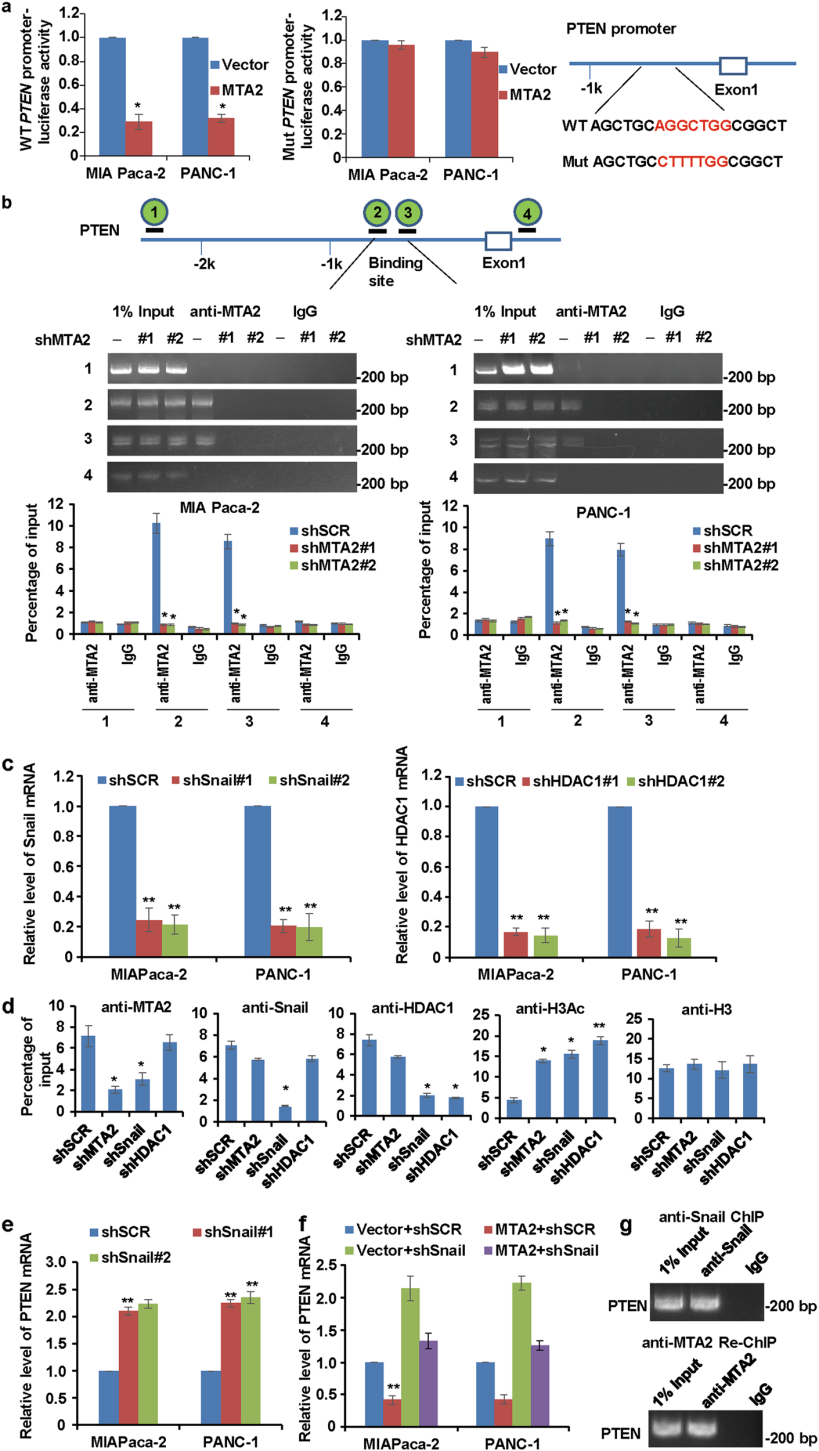
Repression of PTEN by MTA2 is dependent on Snail. **a** The luciferase reporter assay in HEK-293T cells co-transfected with the wild-type (WT) or mutant (Mut) *PTEN* promoter luciferase reporter and the vector or *MTA2* constructs. Schematic of the sequence of the putative consensus MTA2-binding element in the human *PTEN* promoter region and the substitution mutations introduced into this binding element sequence are shown. The luciferase reporter activity results were depicted as a bar graph with mean ± S.D. *n* = 3. *P < 0.05. **b** qChIP assays in MIA Paca-2 cells or PANC-1 cells were performed for the presence of MTA2 at the *PTEN* promoter with or without *MTA2* depletion. Diagram of the *PTEN* promoter region with four amplicons used for qPCR analysis. Error bars represent mean ± S.D. *n* = 3. **P < 0.01. **c** Endogenous Snail or HDAC1 level was measured in MIA Paca-2 cells or PANC-1 cells following transduction with *Snail* shRNAs (shSnail) or *HDAC1* shRNAs (shHDAC1) and shSCR (negative control). GAPDH served as an internal control for each group. Values are mean ± S.D. *n* = 3. **P < 0.01. **d** MIA Paca-2 cells were infected with lentiviruses carrying the indicated shRNAs. A qChIP assay was performed using specific antibodies against MTA2, Snail, HDAC1, H3Ac, or H3 to detect their binding onto the *PTEN* promoter. Error bars represent mean ± S.D. for three independent experiments. *P < 0.05; **P < 0.01. **e** qRT-PCR analyses were used to measure the expression of PTEN in MIA Paca-2 or PANC-1 cells transfected with shSCR or shSnail. **f** The expression of PTEN was measured by qRT-PCR in MIA Paca-2 or PANC-1 cells co-transfected with shSnail and the expression construct for MTA2. **g** ChIP and Re-ChIP experiments in MIA Paca-2 cells with the antibodies against Snail and MTA2 or with isotypic IgG as negative controls

To determine whether *PTEN* was a transcriptional target of MTA2, we searched the promoter region of the human *PTEN* gene and identified one site in the promoter region that matched the consensus MTA2-binding element. Luciferase reporter assay showed that the wild-type *PTEN*-luciferase reporter but not the mutant *PTEN*-luciferase reporter could respond to MTA2 (Fig. 4a). ChIP analysis further revealed that endogenous MTA2 protein only bound to the consensus MTA2-binding element sites (amplicons 2 and 3) on the *PTEN* promoter but not the two control sites (amplicons 1 and 4) in both MIA Paca-2 and PANC-1 cells. Introducing *MTA2* shRNA completely abrogated the binding of MTA2 to the *PTEN* promoter (Fig. 4b), thereby ensuring the specificity of the anti-MTA2 ChIP signals.

Snail has been reported to repress the *PTEN* promoter during γ-radiation-induced apoptosis^16^. In breast cancer cells, MTA1 could transcriptionally repress the expression of PTEN by recruiting HDAC4 along with the transcription factor Yin-Yang 1 (YY1) onto the *PTEN* promoter^34^. Moreover, Snail is a master regulator for triggering epithelial-to-mesenchymal transition and a necessary factor for the survival of migrating cells in tumors^35,36^. Based on these observations, we hypothesized that the repression of *PTEN* by MTA2 was dependent on Snail. To confirm this inference, we further established the stable *Snail* or *HDAC1* knockdown MIA Paca-2 cells using lentivirus-delivered specific shRNA (Fig. 4c). qChIP analysis showed that when *Snail* was depleted, the recruitment of both MTA2 and HDAC1 to the *PTEN* target promoter was dramatically reduced (Fig. 4d). Although depletion of *MTA2* or *HDAC1* resulted in only a marginal effect on the recruitment of Snail to *PTEN* target promoter, their catalytic activities appeared to be interdependent (Fig. 4d). Next, we performed loss-of-function studies using transfection of *Snail* in MIA Paca-2 and PANC-1 cells, the expression levels of PTEN were markedly increased in the two cell lines after *Snail* knockdown (Fig. 4e). Moreover, experiments with *Snail* depletion indicated that the repression of PTEN by overexpression of *MTA2* was, at least partially, dependent on Snail (Fig. 4f). To further support the proposition that Snail recruited MTA2 to form one protein complex at *PTEN* promoters, sequential ChIP or ChIP/Re-ChIP experiments were performed, soluble chromatins were first immunoprecipitated with anti-Snail antibody, the immunoprecipitates were subsequently re-immunoprecipitated with anti-MTA2 antibody. The results showed that in the precipitates, the *PTEN* promoters that were immunoprecipitated with anti-Snail antibody could be re-immunoprecipitated with anti-MTA2 antibody (Fig. 4g). These data suggested that Snail could recruit MTA2 to target *PTEN* promoter and thus inhibit the expression of PTEN.

### MTA2 promotes the proliferation of PDAC cells in vitro and the growth of PDAC xenograft tumor in vivo through inhibition of PTEN

**Fig. 5:**
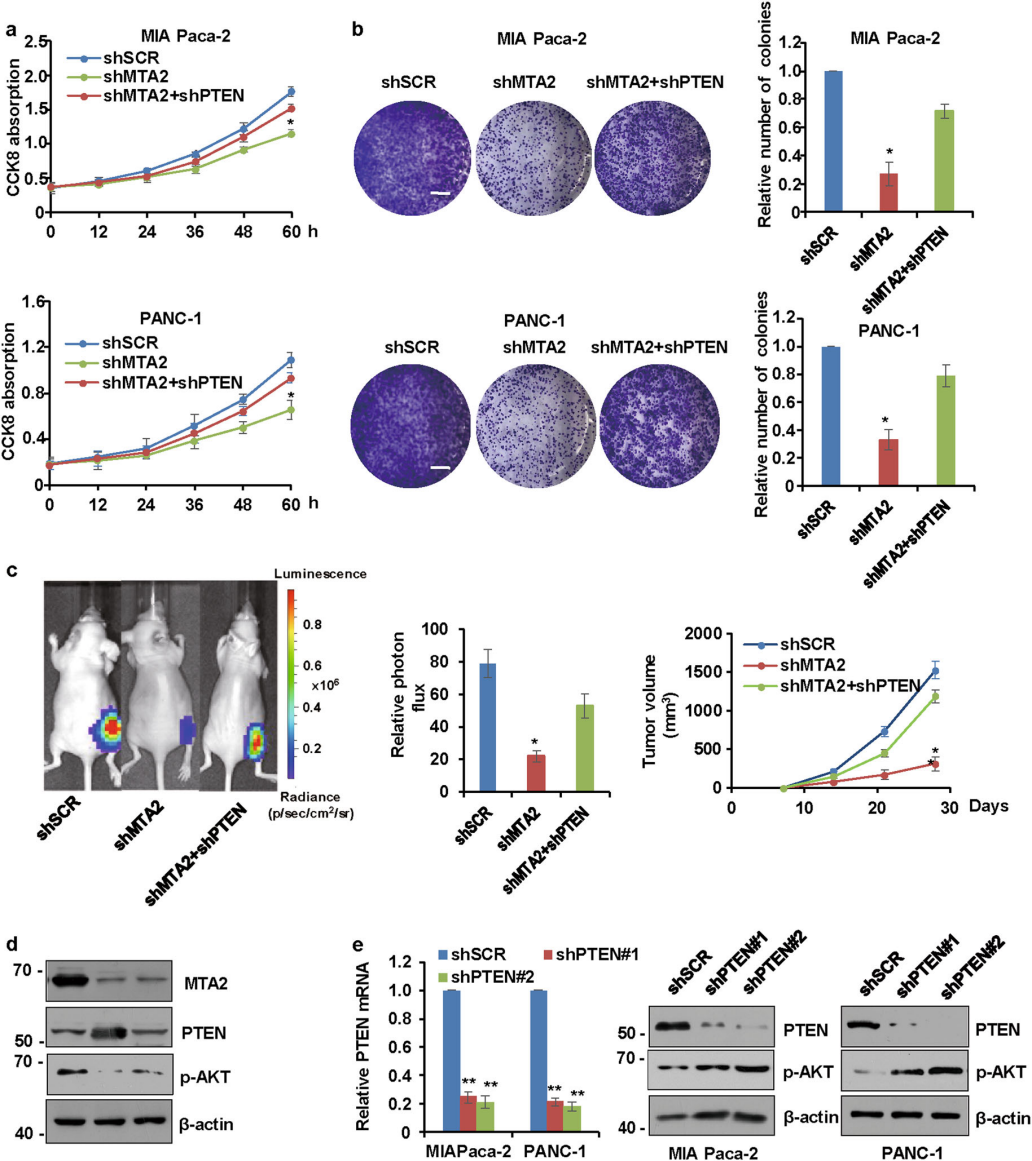
MTA2 promotes the proliferation of PDAC cells in vitro and the growth of xenograft PDAC tumor in vivo through inhibition of PTEN. **a** CCK-8 assays were performed to examine cell proliferation in MIA Paca-2 or PANC-1 cells infected with shSCR, shMTA2, or shMTA2 plus *PTEN* shRNA (shPTEN), respectively, at the indicated time points. Data are expressed as mean ± S.D. *n* = 3. *P < 0.05. **b** Colony formation assays were performed in MIA Paca-2 or PANC-1 cells infected with shSCR, shMTA2, or shMTA2 plus shPTEN, respectively. Values are mean ± S.D. *n* = 3. *P < 0.05. **c** Xenograft tumors were quantified using bioluminescence imaging and tumor volume was weekly measured for 4 weeks after initial implantation with MIA Paca-2 cells. Representative in vivo bioluminescent images at the end of 4 weeks are shown (left) and the tumors were examined by bioluminescent measurement (middle), and tumor volume was also calculated at the indicated time points (right). Data are expressed as mean ± S.D. for three independent experiments. *P < 0.05. Panel **d** indicates protein expressions in isolated tumors at the end of the experiments in **c** which were determined using western blot analysis. **e** qRT-PCR and western blot analyses were used to measure PTEN expression and p-AKT protein levels in MIA Paca-2 or PANC-1 cells infected with shSCR or shPTEN. Values are mean ± S.D. *n* = 3. **P < 0.01

To analyze the function of MTA2 in PDAC, MIA Paca-2 cells or PANC-1 cells were transfected with *MTA2* shRNAs and cell proliferation assays were performed. Our in vitro studies showed that knockdown of *MTA2* significantly decreased the proliferation of MIA Paca-2 cells or PANC-1 cells as indicated by Cell Counting Kit-8 (CCK-8) assay (Fig. 5a) and colony formation assay (Fig. 5b). To assess whether MTA2 also affected PDAC tumor growth in vivo, we injected the *MTA2*-depleted MIA Paca-2 cells with stably expressing firefly luciferase into the right flank of immunodeficient nude mice. Tumor growth was detected by using both quantitative bioluminescence imaging and tumor volume measurement. The *MTA2*-depleted MIA Paca-2 cells showed significantly weakened tumor growth ability 4 weeks after cell implantation (Fig. 5c). Compared with the shSCR group, *MTA2* shRNA could increase PTEN levels and decrease p-AKT levels in the isolated tumor samples (Fig. 5d). To clarify whether knockdown of *MTA2* affected PDAC cell proliferation in a PTEN-dependent manner, we further ectopically inhibited *PTEN* expression with shRNA in PDAC cells. The quantitative reverse transcription polymerase chain reaction (qRT-PCR) and western blot analyses confirmed that when transfected with *PTEN* shRNA, PTEN expression levels in either MIA Paca-2 cells or PANC-1 cells were significantly decreased, while the p-AKT protein level was increased subsequently (Fig. 5e). Next, we assessed the proliferation capacity of cells bearing either individual or compound depletion of *MTA2* and *PTEN*. As shown in Fig. 5a–c, inhibition of *PTEN* could significantly eliminate the blunted proliferation capacity of MTA2-deficient cells, suggesting that MTA2 affected the PDAC cell proliferation and PDAC xenograft tumor growth via a PTEN-mediated mechanism.

### MTA2 enhances the potential of migration and invasion, and activates the PI3K/AKT signaling in PDAC cells in a PTEN-dependent manner

**Fig. 6:**
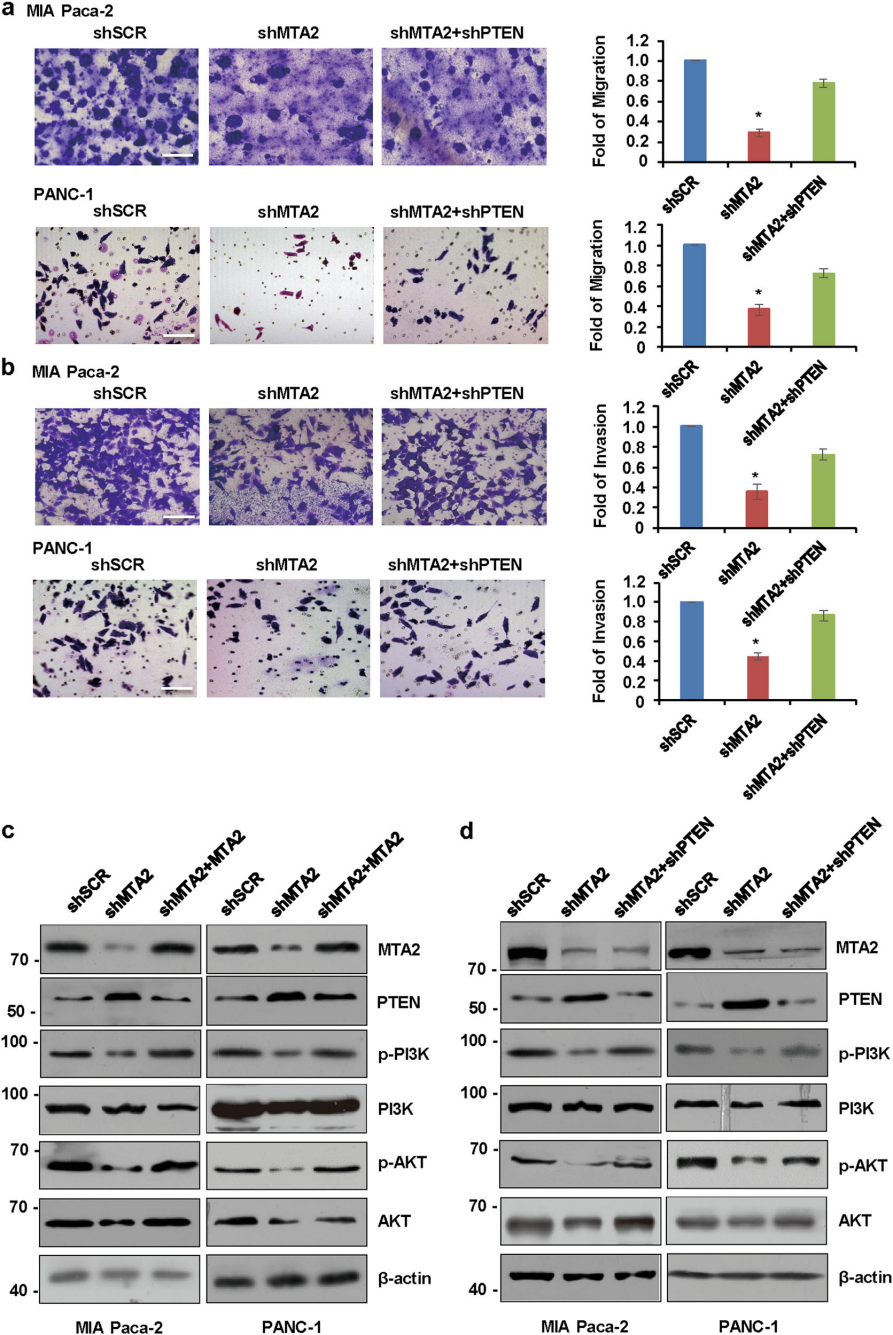
MTA2 enhances the potential of migration and invasion, and activates the PI3K/AKT signaling in PDAC cells in a PTEN-dependent manner. **a**, **b** MIA Paca-2 or PANC-1 cells were infected with shSCR, shMTA2, or shMTA2 plus shPTEN, respectively. **a** Cell migration or **b** invasion was determined using a transwell migration or invasion assay, respectively. Representative images (magnification, ×20) are shown. Data are presented as mean ± S.D. for at least three independent experiments. *P < 0.05. **c** Western blot analysis was used for investigating endogenous phosphorylation levels of the PI3K and AKT following MTA2 inhibition in MIA Paca-2 or PANC-1 cells, and the additional introduction of *MTA2* was used for the rescued assay. **d** Western blot analysis was used for investigating endogenous phosphorylation levels of the PI3K and AKT in *PTEN*-knockdown MIA Paca-2 or PANC-1 cells following MTA2 inhibition

When we monitored the invasive potential of cells upon *MTA2* knockdown, we noticed that ectopically inhibited *MTA2* in MIA Paca-2 cells or PANC-1 cells significantly blunted the cell migration (Fig. 6a) and invasion (Fig. 6b). Further depletion of *PTEN* markedly restored the migration and invasion abilities of the *MTA2* knockdown cells (Fig. 6a, b). Next, we strengthened our findings by suppressing PTEN expression to determine if the MTA2-mediated PTEN inhibition might affect PI3K/AKT signaling in the PDAC cells. Compared with the control cells, a reduced activation of the PI3K/AKT pathway, as indicated by a decreased phosphorylation of the p85 subunit of PI3K and AKT, was observed in the *MTA2* knockdown PDAC cells (Fig. 6c), presumably because of increased PTEN expression. To rule out any potential off-target effects of *MTA2* shRNA, we performed rescue experiments in *MTA2*-depleted cells by adding back a shRNA-resistant form of *MTA2*. Consistently, the noticed defect of the PI3K/AKT signaling in the *MTA2* knockdown cells could be rescued with the introduction of *MTA2* (Fig. 6c). To fortify our findings that MTA2 affected the PI3K/AKT signaling activity by suppressing PTEN expression, we further examined the effect of *PTEN* knockdown in MTA2-deficient cells. Resumed activation of the PI3K/AKT pathway was observed after *PTEN* knockdown in *MTA2*-depleted cells (Fig. 6d).

### BITC inactivates the PI3K/AKT signaling through inhibition of MTA2 in PDAC cells

**Fig. 7:**
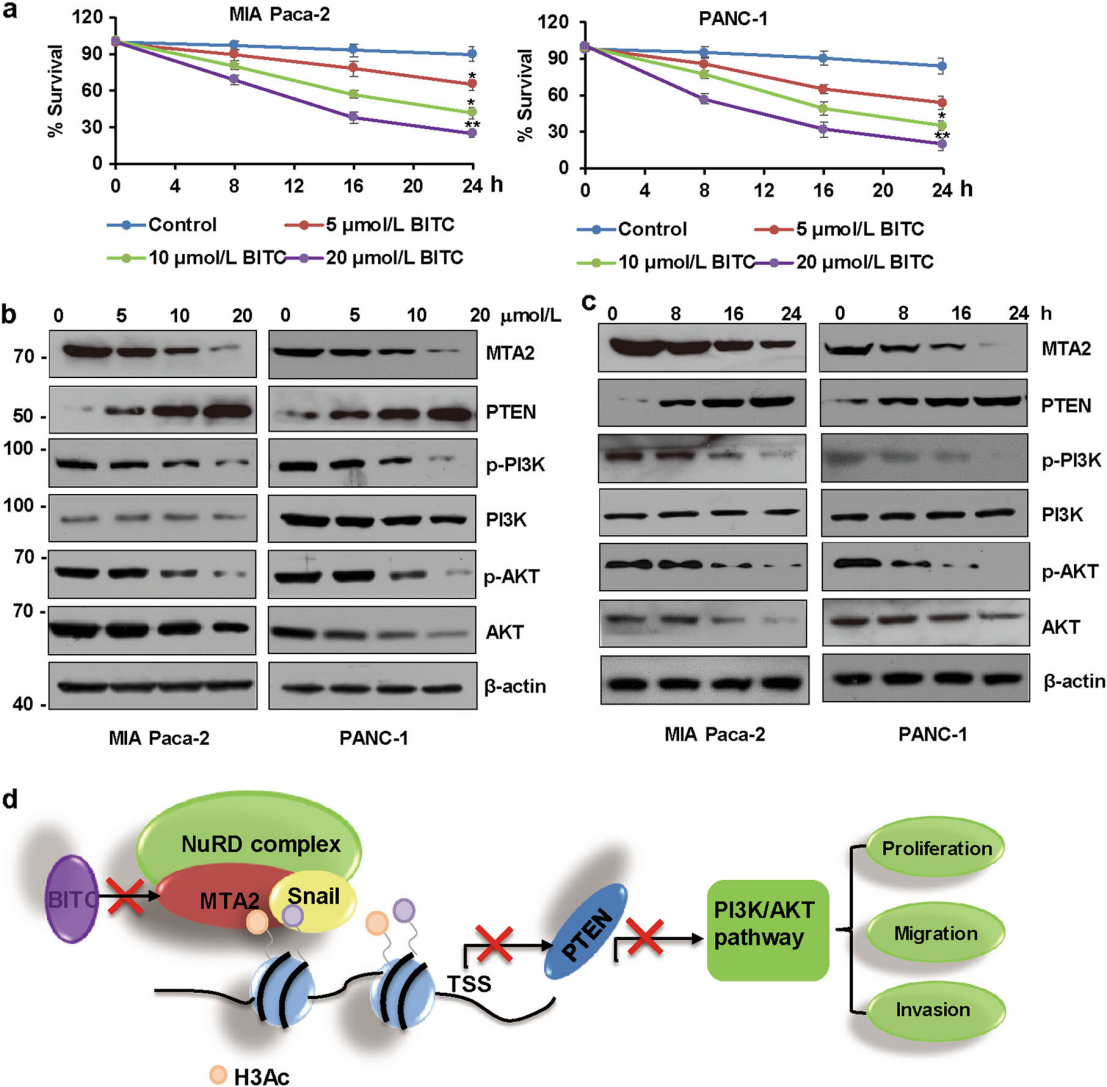
BITC inactivates the PI3K/AKT signaling pathway through inhibition of MTA2 in PDAC cells. **a** MIA PaCa-2 or PANC-1 cells were treated with varying concentrations of benzyl isothiocyanate (BITC) or dimethyl sulfoxide (as a vehicle control) for 0, 8, 16, and 24 h. Cell proliferation was evaluated by CCK-8 assays. Data are expressed as mean ± S.D. *n* = 3. *P < 0.05, **P < 0.01. **b** MIA Paca-2 or PANC-1 cells were treated with different concentrations of BITC for 24 h, and the whole-cell lysates were subjected to western blot analysis. The protein levels of MTA2, PTEN, phosphorylated PI3K (p-PI3K), PI3K, p-AKT, and AKT were measured. **c** MIA Paca-2 or PANC-1 cells were treated with 10 μmol/L BITC for the specified durations. The protein levels of MTA2, PTEN, p-PI3K, PI3K, p-AKT, and AKT were measured using western blot. **d** Proposed model for the role of MTA2 in PDAC cells. *PTEN* is identified as one of the genomic targets of MTA2/NuRD, and PTEN suppression by MTA2 is recruited by Snail. The mechanism of MTA2-mediated PDAC cell proliferation, migration, and invasion is due to the noted repression of PTEN and activation of the PI3K/AKT signaling pathway. In addition, *MTA2* works as the molecular target of BITC in the human pancreatic cancer cells

The antineoplastic agent BITC has been extensively investigated as an anticancer therapy by targeting multiple pro-oncogenic pathways^28,37^. In the present study, we found that BITC could inhibit the proliferation of both MIA Paca-2 and PANC-1 cells in a time- and dose-dependent manner (Fig. 7a). Notably, BITC has been reported to suppress the proliferation of human pancreatic cancer cells via inhibition of the PI3K/AKT/FOXO pathway^38^. That finding prompted us to investigate whether BITC had any inhibitory effects on MTA2 levels directly. We treated MIA Paca-2 cells and PANC-1 cells with varying concentrations of BITC for 24 h. We found a significant downregulation of MTA2 upon BITC treatment in a dose-dependent manner (Fig. 7b). In a time-kinetic study, 10 μmol/L BITC decreased the expression level of MTA2 as early as 8 h after the treatment and continued until 24 h (Fig. 7c). Importantly, the BITC-mediated downregulation of MTA2 levels were concomitant with an upregulation of PTEN level and accompanied by a decreased phosphorylation of PI3K and AKT in either MIA Paca-2 cells or PANC-1 cells (Fig. 7b, c), suggesting that BITC downregulated the PI3K/AKT signaling through inhibition of MTA2. Notably, *MTA2* overexpression or *PTEN* knockdown confers resistance to the growth-suppressive effects of 10 μmol/L BITC but not 20 μmol/L BITC in the pancreatic cancer cells (Supplementary Figure 2). Taken together, these results established a critical role of MTA2 in the BITC-mediated PDAC growth suppression through PTEN.

## Discussion

In our bioinformatic analysis using several databases, the high expression of MTA2 was noticed in the PDAC tissues compared with the normal pancreatic tissues. Kaplan–Meier survival analysis showed that a higher expression level of MTA2 was associated with a poorer overall survival in patients with PDAC. Specifically, our immunohistochemistry study showed that the expression of MTA2 was positively strong in PDAC TMA specimens. In addition, further analyses using both TCGA database and the PDAC TMAs revealed that the high expression of MTA2 was associated with adverse clinical features of PDAC patients. In our in vitro study, knockdown of *MTA2* significantly inhibited the proliferation, migration, and invasion of PDAC cells. Meanwhile, xenograft tumor model study also showed that knockdown of *MTA2* inhibited the tumor growth in vivo. Using ChIP-seq analysis, we identified *PTEN* as a potential target for MTA2. Notably, a strong association between increased MTA2 mRNA expression and decreased PTEN mRNA expression was noticed in the human pancreatic cancer tissues from the online public PDAC databases. Thereafter, we carried out luciferase reporter, qChIP, qRT-PCR, and western blot assays, and found that MTA2 directly bound to the promoter of *PTEN* to suppress its expression and that MTA2 could activate the PI3K/AKT signaling through the inhibition of PTEN. Moreover, treatment with BITC, an antineoplastic agent, led to a dose- and time-dependent decrease of MTA2 level with concomitant upregulation of PTEN and downregulation of PI3K/AKT signaling. According to our working model, MTA2, a core subunit of NuRD complex, can be recruited by the transcription factor Snail. Through repression of the PTEN expression, MTA2 promotes the proliferation, migration, and invasion of PDAC cells, thereby leading to the progression of PDAC. Moreover, inhibition of PTEN by MTA2 results in hyperactive PI3K/AKT signaling, which can be repressed by the BITC treatment (Fig. 7d). Therefore, MTA2 can act as an oncogene in PDAC carcinogenicity, which may be considered as a potential prognostic indicator or even a potential therapeutic target in patients with PDAC.

The PI3K/AKT signaling pathway has been shown to play an important role in cell proliferation, differentiation, and migration. A close relationship between MTA1, which is the most studied member of the MTA family and possesses a 63% structure identity of MTA2^39^, and the PI3K/AKT signaling pathway has been observed earlier^34,40^. Moreover, knockdown of *MTA2* in human glioma cells could modulate the expression of the AKT-regulated downstream genes, such as p21 and p53^41^, suggesting a possible interaction between MTA2 and the PI3K/AKT signaling pathway. In the present study, we showed for the first time that through the inhibition of PTEN, MTA2 could activate the PI3K/AKT signaling pathway in either MIA Paca-2 or PANC-1 cells, and thus promote the proliferation, migration and invasion of the PDAC cells.

It is well known that PTEN, the second most mutated tumor suppressor at their genomic level, is a phosphatase that regulates the levels of PIP3 (phosphatidylinositol-3,4,5-trisphosphate). Absence of nuclear PTEN is strongly associated with a high rate of tumorigenesis and a poor prognosis^42–44^. PTEN is frequently suppressed or mutated in a variety of human cancers. Notably, using the cBioportal website, we analyzed 1034 pancreatic adenocarcinoma samples in five cohorts, and noticed that in PDAC, only 0.9% samples had *PTEN* deletion or mutation (https://www.cbioportal.org/). Therefore, the transcriptional suppression of *PTEN* by MTA2 may be a key mechanism in regulating PDAC tumor growth.

Batra et al.^45^ reported that in the BxPC-3 PDAC cells, BITC could cause a significant decrease in the expression and activity of HDAC1, which usually works as a subunit of NuRD complex. In this context, we also provided evidence that BITC specifically inhibited the expression of MTA2, another subunit of the NuRD complex, which further implies the function of BITC in PDAC cell growth might be due to targeting the MTA2/NuRD complex.

The recruitment of MTAs to specific promoter regions, or specific interactions with other transcription factors, leads to transcriptional silencing of their target genes. For example, Snail and SLUG could bind to the E-boxes in the promoter of *E-cadherin* and recruit MTA1 to suppress E-cadherin expression^46^. Escriva et al.^16^ reported that Snail was consistently associated with and directly repressed the *PTEN* promoter. In the present study, we found that Snail also recruited MTA2 to suppress the expression of PTEN in the human pancreatic cancer cells.

It has been reported that in prostate cancer cells, there is an inverse correlation between MTA1 and PTEN, as MTA1 is a negative regulator of *PTEN* via modulating the deacetylation and inactivation of *PTEN*^47^. In the present study, we showed that MTA2 was a negative regulator of *PTEN*, albeit the mechanism was distinctive. ChIP-seq and luciferase reporter analyses revealed that the binding of MTA2 onto the promoter of *PTEN* and that *PTEN* was transcriptionally repressed by MTA2. Furthermore, we also noticed that the level of H3Ac was significantly increased when MTA2 was depleted. Mi-2, MTAs, and HDAC1/2 represent the compulsory components of the NuRD complex^48–50^. Our findings together with previous observations indicate that the function of MTA2 is dependent on the interdependent HDAC catalytic activities^51^. In addition to the main repression function of MTA2, as a subunit of NuRD complex, it can also have alternative potential as a transcriptional activator^52^. In the present study, we demonstrated that MTA2 acted as a transcriptional repressor of *PTEN* in the human pancreatic cancer cells.

In summary, our study shows that MTA2 promotes PDAC cell proliferation, migration, and invasion via a transcriptional repression of *PTEN* and a subsequent activation of the PI3K/AKT signaling pathway. Moreover, inhibition of MTA2 by BITC is associated with an anti-proliferative effect of this agent on PDAC cells. Our findings indicate that MTA2 may be a potential anti-PDAC therapeutic target.

## Materials and methods

### Cell culture, transfection, and treatment

The human pancreatic cancer cell lines MIA Paca-2 and PANC-1, and a normal immortalized human pancreatic duct epithelial cell line HPDE6c7, were obtained from the American Type Culture Collection (ATCC, Manassas, VA, USA). MIA Paca-2 and PANC-1 cells were maintained in Dulbecco’s modified Eagle’s medium (DMEM; Gibco, Carlsbad, CA, USA) and supplemented with 10% fetal bovine serum (FBS; HyClone, Logan, UT, USA), 100 U/mL penicillin, and 100 µg/mL streptomycin (Sigma-Aldrich, St. Louis, MO, USA) in 5% CO_2_ humidified atmosphere at 37 °C. HPDE6c7 cells were cultured in a keratinocyte serum-free medium supplemented with an epidermal growth factor and bovine pituitary extract (Life Technologies, Carlsbad, CA, USA). After cells grew to 60−80% confluence, they were transfected with Lipofectamine 2000 according to the manufacturer’s protocol. Lentivirus-based control shRNA and specific targeting shRNAs were purchased from Sigma-Aldrich. Puromycin (0.5 μg/mL) was used to select stably transduced cells.

For BITC treatment, MIA Paca-2 or PANC-1 cells were treated with 5, 10, and 20 µmol/L BITC or with 0.1% dimethyl sulfoxide (as a vehicle control) for 0, 8, 16, and 24 h, and then CCK-8 assay or western blot analysis was performed accordingly.

### RNA extraction and quantitative real-time PCR analysis

Total RNA was extracted from relative cells using a TRIzol reagent (Invitrogen, Carlsbad, CA, USA) according to the manufacturer’s instructions. RNA concentration was measured using a spectrophotometer (Nano Drop® 1000; Thermo Scientific, Waltham, MA, USA). Next, 2 µg RNA in each group was reversely transcribed into cDNA using the RevertAid First Strand cDNA Synthesis kit (Thermo Scientific) according to the manufacturer’s specification. Quantitative real-time analysis was performed using the SYBR Green Master mix (Roche, Indianapolis, IN, USA) on the ABI 7500 PCR system (Applied Biosystems, Foster City, CA, USA).

The PCR conditions were as follows: denaturation at 94 °C for 5 min followed by 30 cycles of the following reactions: 94 °C for 30 s, 58 °C for 30 s, and 70 °C for 30 s. Lastly, the reaction was amplified at 72 °C for 10 min. A fold change of expression was calculated according to the 2^−ΔΔCt^ method, and GAPDH was used as an internal reference. Primer sequences were as follows: *GAPDH*: forward 5′-AGGTCCACCACTGACACGTT-3′, reverse 5′-GCCTCAAGATCAGCAAT-3′; *MTA2*: forward 5′-TGGTTAGACGGATTGAGGAG-3′, reverse 5′-TCAAACTCC CGAGCATTACT-3′; and *PTEN*: forward 5′-GCTGAAGTGGCTGAAGAG-3′, reverse 5′-GCTGGAGATGGTGTATGG-3′.

### ChIP-seq and qChIP assays

Cells were maintained in DMEM supplemented with 10% FBS. Approximately 5 × 10^7^ cells in each group were cross-linked with formaldehyde (1% final concentration), washed with cold phosphate-buffered saline (PBS), lysed in buffer, and sonicated on ice to fragment the chromatin to the average length of 300 to 500 bp. The chromatin DNA was precipitated by either normal rabbit IgG (control) or polyclonal anti-MTA2 antibody (sc-28731; Santa Cruz Biotechnology, Santa Cruz, CA, USA). The protein–DNA complexes were incubated with protein A Sepharose beads, and eluted in 1% SDS/0.1 M NaHCO_3_. The protein–DNA cross-link was reversed by heating at 65 °C for 6 h. DNA was recovered by phenol–chloroform extraction and ethanol precipitation and then subjected to in-depth whole-genome DNA sequencing (CapitalBio Corporation, Beijing, China). The raw sequencing image data were examined by the Illumina analysis pipeline, aligned to the unmasked human reference genome (NCBI v36, hg18), and further analyzed by MACS (Model-based Analysis for ChIP-Seq). For the qChIP assay, anti-MTA2, anti-Snail (ab82846, Abcam, Cambridge, UK), anti-HDAC1 (34589, Cell Signaling Technology, Danvers, MA, USA), anti-H3Ac (06–599, Millipore, Bedford, MA, USA) and anti-H3 (ab1791, Abcam) antibodies were used, and the enrichment of the DNA template was analyzed by quantitative PCR. ChIP/Re-ChIP was done essentially the same as the primary ChIP. Bead elutes from the first immunoprecipitation were incubated with 10 mmol/L DTT and diluted 1:50 in dilution buffer (1% Triton X-100, 2 mmol/L EDTA, 150 mmol/L NaCl, and 20 mmol/L Tris-HCl (pH 8.1)) followed by Re-ChIP with the secondary antibodies.

### Western blot analysis

The cells were harvested and lysed with RIPA lysis buffer containing protease and phosphatase inhibitor (Roche) on ice for 40 min. After centrifugation at 13,000 rpm for 15 min, the supernatant was collected, and the protein concentration was measured using the BCA kit (Thermo Scientific). Next, 30 μg protein in each group was subjected to 8−12% SDS-PAGE and transferred onto nitrocellulose membranes by electroblotting. The membranes were blocked in Tris-buffered saline with Tween-20 containing 5% skim milk for 1 h at room temperature. The membranes were then incubated with the primary antibodies at 4 °C overnight and incubated with the corresponding secondary antibodies (horseradish peroxidase-conjugated goat anti-rabbit IgG or goat anti-mouse IgG at 1:3000 dilution; Santa Cruz Biotechnology). An enhanced chemiluminescence kit (Amersham Pharmacia Biotech, Amersham, UK) was used to detect the bands.

### Luciferase reporter assay

The wild-type or mutant *PTEN* promoter product was blunted, kinased, and then cloned into the pGL3basic vector (Promega, Madison, WI, USA). The cells were seeded in six-well tissue culture plates. Next, 50 ng of *MTA2* or vector plasmid DNA was used along with 100 ng of wild-type *PTEN* or mutant *PTEN* luciferase construct to transfect the cells. Forty-eight hours after transfection, luciferase activity was determined using a dual-luciferase reporter assay system (Promega), following the protocol of the manufacturer. Luciferase activity was normalized to Renilla luciferase activity. Each experiment was performed in triplicate and repeated at least three times.

### CCK-8 assay

To analyze the cell viability, we performed a CCK-8 assay. MIA PaCa-2 or PANC-1 cells were incubated in a 96-well plate at a final density of 5 × 10^3^ cells/well to allow adherence. After incubation for the specified time, 10 μl CCK-8 reaction solution (Dojindo Laboratories, Kumamoto, Japan) was added to each well. The plates were further incubated at 37 °C for 2 h, and the absorbance was finally determined at 490 nm using a microplate reader.

### Colony formation assay

The colony formation assay was performed to detect the anchorage-independent cell growth. The relative infection cells were seeded into six-well culture plates at a density of 1000 cells per plate. The cells were then incubated for 14 days at 37 °C in an incubator with 5% CO_2_. All colonies were fixed with 4% paraformaldehyde, dried in air, and stained with 0.1% crystal violet. The colonies with >50 cells were counted.

### Cell migration and invasion assays

Transwell assays were used to examine cell migration and invasion. The chambers (24 well, 8 µm) were purchased from Merck Millipore (Darmstadt, Germany). For invasion assay, 50 µl of diluted matrigel (BD Biosciences, Franklin Lakes, NJ, USA) was coated on the filter of the upper chamber. The 2 × 10^5^ treated cells were seeded with serum-free DMEM medium onto the top of the chambers without matrigel for migration assay or matrigel-coated invasion chambers for invasion assay. After that step, 500 μl DMEM containing 10% FBS were added to the lower chamber. After 24 or 48 h, the non-migrating or non-invading cells on the upper layer were removed with a cotton swab, while the migrating or invading cells on the bottom of the filter were stained with 0.1% crystal violet, and photographed under an inverted phase contrast microscope at a magnification of ×20 (Olympus, Tokyo, Japan).

### Mouse xenograft tumor model

BALB/c nude mice were purchased from Charles River (Beijing, China). MIA Paca-2 cells were infected with luciferase lentiviruses to obtain a firefly luciferase stably expression construct (Xenogen Corporation, Alameda, CA, USA), and the stable cells were then infected with lentiviruses carrying the control shRNA, *MTA2* shRNA, or *MTA2* shRNA plus *PTEN* shRNA. The 2.5 × 10^6^ cells in each group were injected into the right flank. For bioluminescence imaging, mice were injected intraperitoneally with 200 mg/g of d-luciferin in PBS. Fifteen minutes after injection, the mice were anesthetized, and bioluminescence was imaged with a charge-coupled device camera (IVIS; Xenogen Corporation). Tumor volume was weekly assessed for 4 weeks. Six animals per group were used in each experiment. All studies were approved by the Animal Care and Use Committee of Peking University Health Science Center.

### Database analysis

The mRNA expression pattern of MTA2 referenced during the study is available in the public repository from the cBioPortal for Cancer Genomics (https://www.cbioportal.org/), GEO database (https://www.ncbi.nlm.nih.gov/gds/), ICGC Data Portal (https://dcc.icgc.org/), and Oncomine database (https://www.oncomine.com/). The clinical features and survival data in patients with pancreatic cancer were obtained from TCGA database (https://portal.gdc.cancer.gov/). Immunohistochemistry images of MTA2 staining were taken from the online database of the Human Protein Atlas (https://www.proteinatlas.org). Immunohistochemistry analysis was carried out as reported previously^30,31^.

### TMAs and immunohistochemistry

The clinical significance of MTA2 expression in PDAC patients was analyzed using TMAs obtained from Xian Alenabio Biotech (Shanxi, China) that contained 64 PDAC tissues and adjacent normal pancreatic tissues. Xian Alenabio Biotech also provided patients’ information, including gender, age, pathological grade, and TNM stage. Use of the TMAs complied with relevant regulations, and was approved by the Ethics Committee of Peking University Health Science Center.

Antigen retrieval was performed by incubating the samples in sodium citrate solution (0.01mol/L, pH 6.0) buffer at high pressure for 15 min. Subsequently, endogenous peroxidase activity was blocked using 3% hydrogen peroxide, the TMAs were incubated with anti-MTA2 antibody (1:20) at 4 °C overnight, and then incubated with secondary antibody for 1 h at room temperature. For immunohistochemistry quantification, all samples were blind scored (from 0, lowest staining intensity, to 3, highest staining intensity) by three independent observers. The average score was calculated for each tumor sample.

### Statistical analysis

Statistical analysis was conducted using SPSS software (version 17.0; SPSS Japan Inc., Tokyo, Japan). Each experiment was repeated at least three times. The data were expressed as mean ± S.D. and analyzed using Student’s *t*-test or one-way ANOVA (followed by the post-hoc Tukey–Kramer test) where appropriate. Statistical analysis of overall survival time was performed using Kaplan–Meier curves of log rank analysis. *P* < 0.05 was considered to indicate a statistically significant result.

## Supplementary information

Supplementary Table 1

Supplementary Table 2

Supplementary Table 3

Supplementary Figures
