# Supplementary material for: MTA2-mediated inhibition of PTEN leads to pancreatic ductal adenocarcinoma carcinogenicity
Source: Cell Death Dis. 2019 Feb 27;10(3):206. doi: 10.1038/s41419-019-1424-5 (PMC6393561; doi:10.1038/s41419-019-1424-5)
Supplement: Supplementary file 1 — Supplementary Table 1 [file 41419_2019_1424_MOESM1_ESM.doc]

**Supplementary Table 1. Patients’ information used in this analysis in the GSE28735, ICGC, TCGA and Badea’s cohorts**

|  | **GSE28735** | **ICGC** | **TCGA** | **Badea’s cohort** |
| --- | --- | --- | --- | --- |
| Normal (No.) | 45 | 110 | 171 | 36 |
| PDAC (No.) | 45 | 189 | 176 | 36 |
