# Supplementary material for: MTA2-mediated inhibition of PTEN leads to pancreatic ductal adenocarcinoma carcinogenicity
Source: Cell Death Dis. 2019 Feb 27;10(3):206. doi: 10.1038/s41419-019-1424-5 (PMC6393561; doi:10.1038/s41419-019-1424-5)
Supplement: Supplementary file 2 — Supplementary Table 2 [file 41419_2019_1424_MOESM2_ESM.doc]

**Supplementary Table 2. Correlations between MTA2 expression level and clinicopathologic parameters in TCGA database**

| **Parameters** | **No.**  **(n = 176)** | **MTA2 expression** | | **χ^2^** | ***P*** |
| --- | --- | --- | --- | --- | --- |
|  |  | **High**  **(n = 96)** | **Low**  **(n = 80)** |  |  |
| Gender | | | | | |
| Male | 96 | 53 | 43 | 0.037 | 0.847 |
| Female | 80 | 43 | 37 |  |  |
| Age (years) | | | | | |
| ≤60 | 58 | 30 | 28 | 0.278 | 0.598 |
| >60 | 118 | 66 | 52 |  |  |
| AJCC Stage | | | | | |
| I-IIa | 52 | 22 | 30 | 4.458 | 0.035 |
| IIb-IV | 124 | 74 | 50 |  |  |

AJCC, American Joint Committee on Cancer
