# Supplementary material for: MTA2-mediated inhibition of PTEN leads to pancreatic ductal adenocarcinoma carcinogenicity
Source: Cell Death Dis. 2019 Feb 27;10(3):206. doi: 10.1038/s41419-019-1424-5 (PMC6393561; doi:10.1038/s41419-019-1424-5)
Supplement: Supplementary file 3 — Supplementary Table 3 [file 41419_2019_1424_MOESM3_ESM.doc]

**Supplementary Table 3. Correlations between MTA2 expression level by tissue microarray and clinicopathologic parameters in pancreatic cancer patients**

| **Variables** | **No.**  **(n=64)** | **MTA2 expression** | | **χ^2^** | ***P*** |
| --- | --- | --- | --- | --- | --- |
|  |  | **Low (n=27)** | **High (n=37)** |  |  |
| Gender |  |  |  |  |  |
| Male | 38 | 17 | 21 | 0.249 | 0.618 |
| Female | 26 | 10 | 16 |  |  |
| Age (years) |  |  |  |  |  |
| ≤ 60 | 49 | 19 | 30 | 0.998 | 0.318 |
| >60 | 15 | 8 | 7 |  |  |
| Pathological grade |  |  |  |  |  |
| I-II | 40 | 22 | 18 | 7.179 | 0.007 |
| III | 24 | 5 | 19 |  |  |
| T stage |  |  |  |  |  |
| T1-T2 | 37 | 21 | 16 | 7.633 | 0.006 |
| T3-T4 | 27 | 6 | 21 |  |  |
| N stage |  |  |  |  |  |
| N0 | 50 | 24 | 26 | 3.166 | 0.141 |
| N1 | 14 | 3 | 11 |  |  |
