# Supplementary material for: MTA2-mediated inhibition of PTEN leads to pancreatic ductal adenocarcinoma carcinogenicity
Source: Cell Death Dis. 2019 Feb 27;10(3):206. doi: 10.1038/s41419-019-1424-5 (PMC6393561; doi:10.1038/s41419-019-1424-5)
Supplement: Supplementary file 4 — Supplementary Figures [file 41419_2019_1424_MOESM4_ESM.doc]

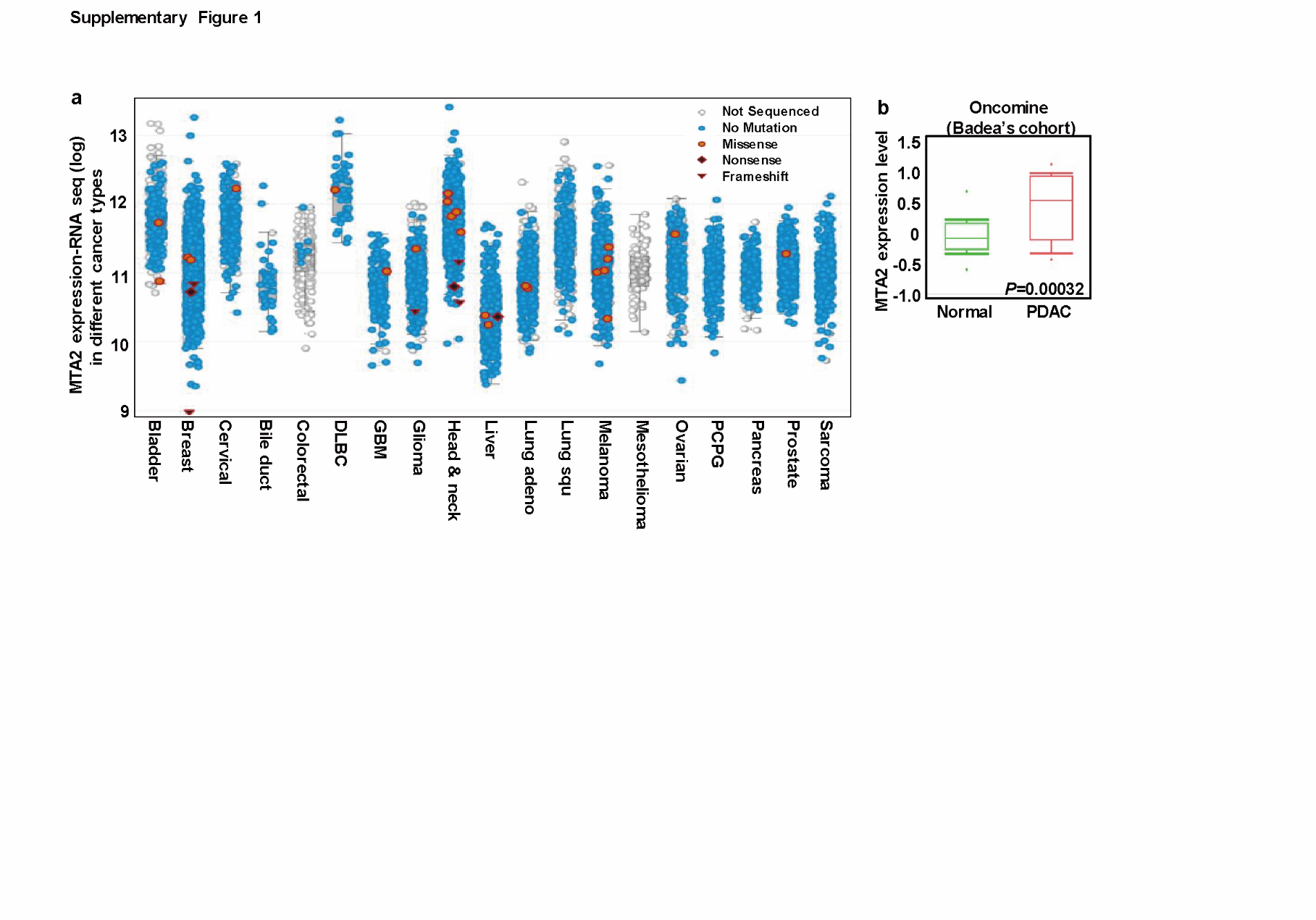
**Supplementary Figure 1. MTA2 is overexpressed in different cancer types including pancreatic carcinoma**

**a** The genomic alterations of MTA2 in several types of cancer, according to the database of cBioPortal for Cancer Genomics. **b** The relative expression of MTA2 was measured in pancreatic ductal adenocarcinoma (PDAC) specimens and normal pancreatic tissues in the Oncomine (Badea’s cohort).


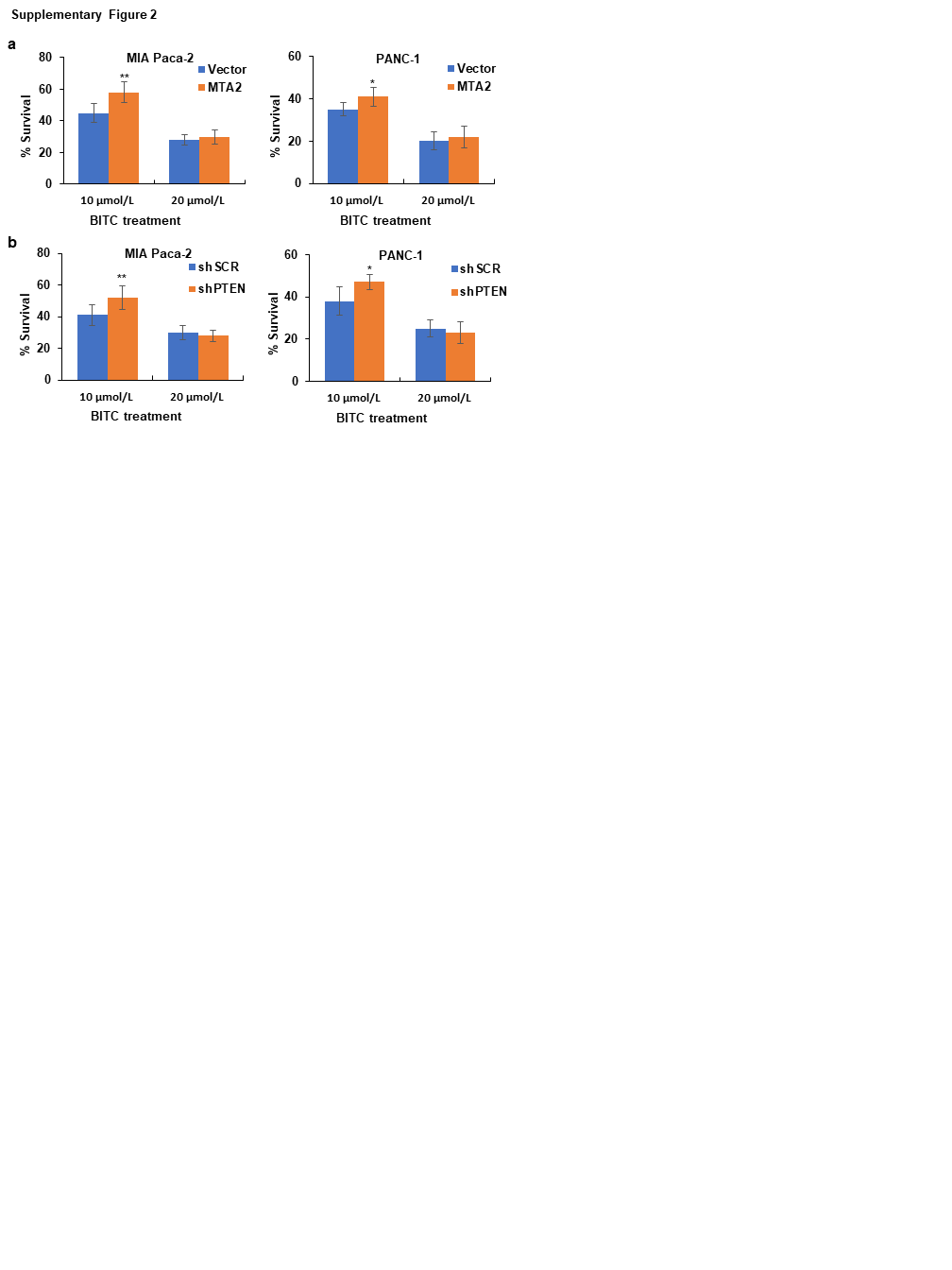


**Supplementary Figure 2. Effects of different concentration of BITC in PDAC cells.**

**a, b** MIA Paca-2 or PANC-1 cells transfected with **a** vector or MTA2 construct, or **b** with shSCR or shPTEN for 48 h followed by treatment with 10 μmol/L or 20 μmol/L BITC for additional 24 h, and the viability was analyzed by CCK-8 assay. Error bars represent mean ± S.D. for three independent experiments. **P*<0.05; ***P*<0.01.
